# Group sparse coding with a collection of winner-take-all networks

**DOI:** 10.1186/1471-2202-13-S1-P184

**Published:** 2012-07-16

**Authors:** Eva L Dyer, Ueli Rutishauser, Richard G Baraniuk

**Affiliations:** 1 Electrical & Computer Engineering Department, Rice University, Houston, TX, USA; 2 Max Planck Institute for Brain Research, Frankfurt, Germany

## 

Degeneracy is a ubiquitous feature of computation and coding in biological systems. *Degenerate codes*—codes in which multiple code words have the same meaning or interpretation—arise in a wide range of biological processes, from the many-to-one mapping of codons to amino acids to the numerous instances of degenerate coding in the nervous system, both in the periphery and in the cortex. There are a number of reasons why neural systems might seek some degree of degeneracy; by enabling a number of inputs (stimuli) to be mapped to the same output code, the system is endowed with a certain level of robustness to noise and to cell death. Furthermore, degenerate codes provide invariance to certain types of variability in the raw sensory input. This is particularly useful in object recognition for instance, where despite photometric variability, the representation of an object under different illumination conditions should be equivalent at some level of processing even if the neural representations for these states are slightly different.

In tandem with nature's desire to produce degeneracy and invariance in the brain’s representation of sensory inputs, *sparsity* also appears to also play an important role in neural coding; a sparse code is one that requires a small number of active neurons relative to the size of the population. Sparse codes are also associated with a high degree of specificity: each cell in the population only responds to a limited number of inputs. Sparse coding has been observed in the visual cortex of macaque, mushroom body in locusts, and auditory cortex in grasshoppers.

Experimental studies suggest that neural systems might seek to strike an appropriate balance between the degeneracy and the sparsity of the neural code. Here, we describe a framework in which one can tradeoff between these two objectives in a natural way. In contrast to models for global sparse coding such as the locally competitive architectures in LCA [1] which find a population code that captures the primary features of the stimulus while minimizing the number of active neurons in the population, our goal is to find a representation of the sensory input that minimizes the number of ‘groups’ that must be active to encode the input. The idea is that when the excitatory cells are grouped in a meaningful way, the activation of a small set of groups might have a particular meaning to the organism. If we view the activation of these ‘functional groups’ as a high-level or coarse scale representation of the stimulus, the resulting code is stable to a wide-range of perturbations in the input space. This two-level representation (the coarse and the fine) provides a robust and degenerate mapping of the input space that is also sparse.

After motivating the utility of group sparse coding in neural systems, we show how one can implement these sparse coding networks by coupling a collection of winner-take-all (WTAs) networks that were first introduced for state-dependent computation in [2]. WTAs are particularly attractive as they represent neurally plausible microcircuits that have been hypothesized to serve as a computational primitive in complex cortical networks. An interesting property of these microcircuits is the fact that a single inhibitory unit delivers a common inhibitory signal to all of the excitatory units within the WTA. This architecture is in stark contrast the highly specified point-to-point inhibition structure required to faithfully implement a LCA, i.e., the inhibition between every pair of cells can be different. To solve sparse coding problems with collections of WTAs, we adapt the analytical approach in [1] and show how one can couple a collection of WTAs to descend an energy function that promotes grouped sparse representations. In addition to building in invariances into the code, we demonstrate that there are a number of computational advantages to employing this type of grouped network model for sparse coding. First, we demonstrate that we require fewer “long-range” connections to produce group sparse codes with collections of WTAs than that required for a global LCA. Second, the lateral and recurrent inhibition in the network modulates the threshold of excitatory units collectively: this means that fewer interneurons are required to produce a representation, the number of long range messages which must be sent is reduced, and the network converges to a solution faster than the global LCA.
